# Smart Query Answering for Marine Sensor Data

**DOI:** 10.3390/s110302885

**Published:** 2011-03-03

**Authors:** Md. Sumon Shahriar, Paulo de Souza, Greg Timms

**Affiliations:** Tasmanian ICT Centre, CSIRO, Hobart, Tasmania 7001, Australia; E-Mails: paulo.desouza@csiro.au (P.D.S.); greg.timms@csiro.au (G.T.)

**Keywords:** marine sensor network, sensor data, smart query

## Abstract

We review existing query answering systems for sensor data. We then propose an extended query answering approach termed *smart query*, specifically for marine sensor data. The smart query answering system integrates pattern queries and continuous queries. The proposed smart query system considers both streaming data and historical data from marine sensor networks. The smart query also uses query relaxation technique and semantics from domain knowledge as a recommender system. The proposed smart query benefits in building data and information systems for marine sensor networks.

## 1. Introduction

With the emergence of inexpensive and smart sensors, many sensor networks are currently being deployed for different scientific purposes such as environmental monitoring, health and energy [1,2]. As the cost of sensors is decreasing rapidly, it is also anticipated that there will be an increasing deployment of sensor networks in the future.

The streaming data generated from sensor networks are usually collected and stored in different database management systems [3,4] for future scientific uses. The raw data generated from sensor networks can also be stored temporarily in network memory or small databases in a distributed approach [5] before being stored permanently. This temporarily stored data is important for real-time stream query processing, quality assurance and quality control (QA/QC), outlier and event detection, and data cleaning. As resources such as energy, cost of communications, memory and processing power are limited for wireless sensor networks, the sensor data are further preferred to be stored in centralized databases for future use such as data and knowledge mining [6].

Marine sensor data come from sensor networks deployed in a marine environment [7,8]. Types of marine environments include estuaries, seas and oceans. Types of marine sensor data include water temperature, pressure (proxy for depth), turbidity, conductivity (proxy for salinity), dissolved oxygen, chlorophyll, images, *etc*.

In most cases, the raw data stored in databases are first retrieved and processed using mathematical and statistical tools (e.g., SPSS, R, Matlab) and are then visualized when needed by the application. In addition to processing and visualizing sensor data using those tools, there is also a need to process queries on sensor data for automatic prediction, awareness and decision making. To add value to the query, there is a need to process both real-time or near real-time streaming data and the stored historical data. In addition to processing data, there is also a need to add semantics when answering the query. We term this query answering system *smart query*.

**Figure 1.**
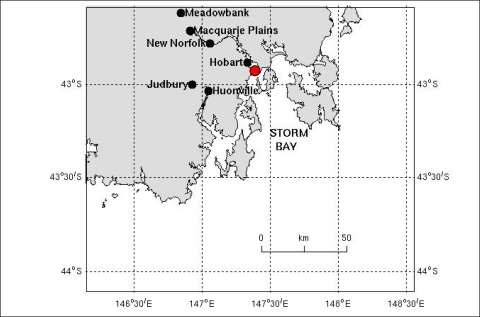
Query region (South East Tasmania, Australia).

As types of marine sensor data are diverse, query processing is a challenging task in the sense that there may be correlation among different phenomena. Besides, there is a need to transform and integrate sensor data for data mining and knowledge discovery processes. We provide some motivating examples in the context of the region shown in Figure 1.

Query 1: Will the dissolved oxygen decrease by 10% tomorrow in the region?

This query can not be answered directly from databases. There is a need to process both real-time and historical marine sensor data and other domain data (e.g., weather) for prediction and then to answer the query.

Query 2: Find the best location for fishing on next Sunday in the Derwent river at Hobart.

This query can not be answered simply using traditional query languages. It requires some knowledge of marine sensor databases. The knowledge can be derived from sensor data [5] with different phenomena such as water temperature and water quality that affect the fish movement and the fish abundance for different locations in the Derwent river.

Query 3: Find the best location for surfing this summer at South East Tasmania.

This query is similar to the previous query but it requires tide and wind data as well as information from marine sensor data.

This review paper is organized as follows. In Section 2, we first give an overview of traditional databases and intelligent query processing in traditional databases. We then review traditional query processing for sensor databases and the sensor web in Section 3. The system components of the proposed smart query system are described in Section 4. The research problems and challenges of smart query processing for marine sensor data are then discussed in Section 5. We give discussions in Section 6 and conclusions in Section 7.

## 2. Databases and Query Answering

In databases (DBs), query answering means to retrieve data according to the requirement of users. The data retrieved from the databases are expected to be exactly on what the users want. In different databases such as relational databases, object-relational databases, semi-structured and XML databases, query languages exist. These query languages, based on query algebra, are used in processing and answering queries.

### Intelligent Query Answering

In database management systems (DBMSs) and information systems (ISs) applications, to get the exact answers from a query, users must have sufficient knowledge of the data stored in the databases. However, in some cases, users may not have sufficient knowledge of the databases to make a successful query. To assist users in answering queries, the systems need to harvest knowledge from the databases. The process of adding knowledge in query answering is called *intelligent query answering* [9,10]. Different techniques are used in answering queries intelligently. Different knowledge discovery tools use different mechanisms such as generalization [9] and data mining [11,12]. Moreover, intelligent query answering can be based on a specific feature such as location [13]. With the emergence of the semantic web [14], location based intelligent query answering using the semantic web [15,16] is also of research interest.

## 3. Query Processing in Sensor Databases and Sensor Web

In the physical world, a massive number of sensors, either wired or wireless [2,17] are currently being deployed in different sectors such as weather, environment, agriculture, fisheries, energy industries, homeland security and health care. Each sensor network that is a collection of different types of sensors is producing a huge amount of data in real time. The produced data are being stored in different scientific data formats (e.g., HDF, DDX, NetCDF, CTD) and in different relational (e.g., PostgreSQL, Oracle) and semi-structured (XML) databases. The databases that store sensor data are termed *sensor databases*.

### 3.1. Query Answering in Sensor Databases

**Figure 2.**
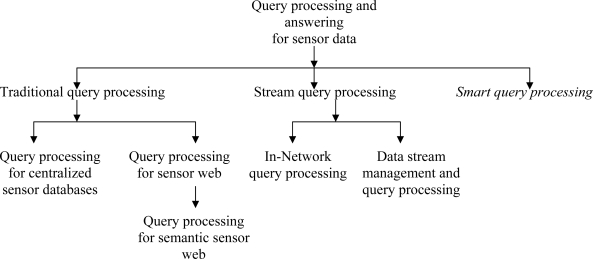
Classification of query processing based on sensor data storage.

Sensor databases [3] store data with different dimensions (e.g., spatial, temporal) and phenomena (e.g., temperature, pressure, humidity). Therefore, in a database or data-centric perspective, processing data from sensor databases [4] is a great challenge. Some data management issues in sensor databases include data exploration and analysis, query processing, data transformation and integration, data mining, data provenance, data interoperability, and data visualization [2]. In case of query processing in sensor databases, there are two ways to approach the problem. Firstly, the query can be posed to centralized databases (*traditional* query) [18]. Secondly, the query can be distributed to the sensor network where there are memories or databases with limited storage and data processing facility [19]. The latter approach is known as *stream* (in-network) query processing. These two approaches of query processing based on data storages for sensor data are shown in Figure 2. In the figure, we also note our proposed smart query processing that includes the properties of both traditional and stream query processing that will be discussed shortly.

As well as different approaches to query processing based on sensor data storage, there are different types of query processing based on types of answers the queries return to satisfy users. There are two types of query processing and answering techniques: *exact* and *approximate*. In the exact query answering technique, the query processing returns the exact discovered values to the users based on the query parameters. On the other hand, in the approximate query answering technique, the close or similar types of answers are returned to the users. In some cases, the users are also given facilities to *relax* the query parameters so that the query returns satisfied answers. We show some examples of exact and approximate query answering techniques in Table 1.

### 3.2. Query Answering in Sensor Web

The diverse types of sensor networks have necessitated the need of a sensor web [20,21] for data interoperability. As a result, different query mechanisms using Sensor Observation Service (SOS) have emerged.

### 3.3. Query Processing in Semantic Sensor Web

Although, the query processing using SOS has fulfilled some aspects of data interoperability, still most semantics can not be captured through sensor web. Thus, with semantic web [14], the semantic sensor web [22] has emerged. Again, query processing on the semantic sensor web [23] is also an important research issue.

## 4. Smart Query Answering System

**Figure 3.**
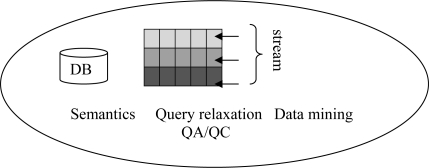
Smart query system components.

We show the examples of existing query answering systems for sensor data in Table 1. In the table, we also show the proposed smart query system. We now describe the components of the smart query system shown in Figure 3. The proposed smart query system considers both historical and streaming data together from marine sensor network. We formulate a pattern query in historical data a to extract similar patterns. Then similar patterns can be used for predictive pattern in forecasting. The similar patterns also exhibit similar events that happened in the past. Besides pattern queries on historical data, continuous queries are formulated on the streaming data to find event patterns similar to the stored patterns in the historical data. The event patterns are used in situation awareness and decision making.

To process queries either for historical data or for streaming data, we propose to augment with query relaxation techniques, data mining methods, QA/QC and semantics for marine sensor data. The query relaxation technique allows users to pose queries with flexibility. Moreover, the query relaxation technique considers query intention. By query intention, we mean that the user may be interested in very close related items. For example, if a user wants to know the water temperature, the user may also be interested in salinity as well in the marine domain.

In the case of data mining, we use similar pattern search algorithms and clustering techniques with QA/QC for query processing.

Although semantics are used mostly for historical data or for a snapshot of data, for streaming data there is currently little use of semantics to the best of our knowledge. Thus we propose to use semantics not only for historical data but also for streaming data for reasoning towards query processing and answering.

We now provide some research problems for our proposed smart query answering with marine sensor data.

## 5. Research Challenges for Smart Query Answering in Marine Sensor Data

**Figure 4.**
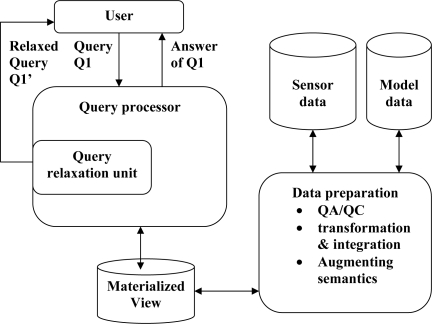
The steps in proposed smart query processing.

In answering a smart query of marine sensor databases, we have identified some issues to be considered. We now illustrate those issues. The steps needed for our proposed smart query processing system are shown in Figure 4.

### 5.1. Data Preparation

Data preparation means pre-processing data for query processing and answering. Marine sensor data can be missing or incomplete. It can also have errors or noise. Besides, different data sources can be in different data models/formats with different phenomena. Thus data need to be processed and prepared before processing and answering a smart query. There are different tasks that need to be incorporated to the data sources for data preparation. We identify the followings:

Data quality and data cleaning: Quality marine sensor data [27] are necessary for efficient smart query processing. Missing or erroneous data affect query processing and answering. Different data mining techniques such as outlier detection need to be considered in measuring data quality. In many applications, data also needs to be cleaned [28,29] before processing queries.

Data aggregation: After cleaning marine sensor data, cleaned data need to be grouped [4] for query processing.

**Figure 5.**
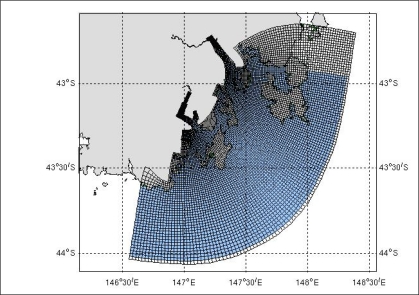
Model grid (South East Tasmania, Australia).

Data transformation and data integration: As marine sensor data is represented and stored in different data formats or models, there is a need to transform data for query processing. For example, the Hydro Dynamic model data [30] shown in Figure 5 are stored in NetCDF format and the real measurement data from a marine sensor network are stored in relational databases. Data from different sensor networks or domains [31] such as Hydrology and Weather also need to be integrated for smart query processing [32].

### 5.2. Discovery of Knowledge and Data Mining Techniques

As the raw marine sensor database contains mainly spatial and temporal data, the answer to the query may not have an exact result because data may not exist in the specified time and space. Thus there is a need to use statistical methods such as interpolation and extrapolation [33–35] for gap filling, forecasting, identifying trends or prediction. Moreover, there may be no exact answer for a query that asks for interesting patterns or behaviors. The user is not interested in the exact solution but the approximate solutions such as identification of similar patterns [36]. In that case, data mining techniques such as pattern extraction and machine learning [6,37] can be exploited. We give a motivating example. Consider a query as the following:

Q: “Find similar patterns of water temperature as presented at location ‘CMAR Wharf’ within South East Tasmania within the last two years.”

**Figure 6.**
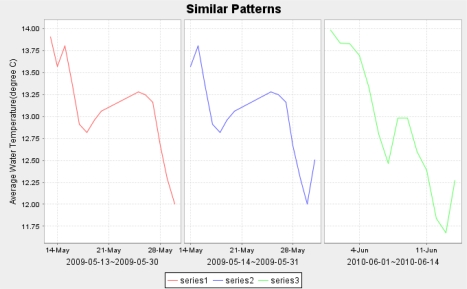
Pattern query.

The result of the query is shown in Figure 6. In this case, pattern mining techniques such as dynamic time warping (DTW) are used.

### 5.3. Event Processing with Continuous Queries

Besides knowledge discovery through pattern query, there is also a need to detect events from streaming sensor data using continuous queries. Event detection will be used for situation awareness and decision making processes in the marine sensor network.

### 5.4. User Modeling and Context Profiling

User profiling: In smart query answering, profiling of users is an important issue. Different users have different expectations from the data and information system.

Context profiling: Context of the query can be characterized by the location [13] and the time of the query.

### 5.5. Query Profiling and Extension

Query profiling: Profiling a query means to identify different types of queries such as *continuous queries* and *snap shot* or *historical queries* [38,39].

Query intention and relaxation: Based on the users, context and query types, query intention can be analyzed and then the query can be relaxed [9].

For example, the query *Q* may be relaxed to discover hidden patterns that may be of interest for the user.

*Q̅: “Find similar patterns of water temperature as presented at ‘CMAR Wharf’ within South East Tasmania within the last* **three** *years”*

In case of query intention, we can extend the query *Q̅* to the following.

*Q̿: “Find similar patterns of* **water temperature and dissolved oxygen and their relationships** *as presented at location ‘CMAR Wharf’ within South East Tasmania within the last* **three** *years.”*

### 5.6. Semantics in Marine Sensor Web

With the emergence of the semantic web, there is a need to extend smart query processing on the marine sensor web to the marine sematic sensor web [40]. The concept of distributed ontology [41] can be used in smart query processing over the marine semantic sensor web. As well, smart queries using semantic reasoning techniques [42,43] can also be developed over the marine semantic sensor web. Consider the following query.

Q: “Publish the ocean event (e.g., AlgalBloom) at the region ‘Huon’ in South East Tasmania.”

This continuous query can be answered using distributed semantic reasoning over the marine sensor web.

We identify the following issues in smart query answering in the marine semantic sensor web.

- Adding ontologies to the marine sensor web.
- Reasoning over the integrated semantic sensor web.
- Distributed query processing over the integrated semantic sensor web.

## 6. Discussions

Sensor networks have already proven to be useful in many areas such as environmental monitoring [44,45], agriculture and water monitoring [46,47], ecosystem monitoring [31,48] and coastal monitoring [21,49].

With the advancement of cheap and smart sensors, a lot of sensor networks are being deployed in the marine environment. As a result, a massive amount of marine sensor data are generated and stored in repositories. Marine sensor data need to be processed, analyzed, represented and transformed to information and knowledge for different purposes such as data publishing [50], situation awareness [42] and data sharing [51]. The information and knowledge obtained from raw marine sensor data needs to be available to answer queries smartly and intelligently. Real time query processing [39] is also necessary as a lot of streaming data are coming from different sensor networks.

## 7. Conclusions

We first reviewed traditional intelligent query processing and intelligent query processing on the semantic web. We further reviewed different existing query processing systems in sensor networks, sensor databases and the semantic sensor web. An extended query processing technique termed *smart query* is proposed using both historical and streaming for marine sensor data. The proposed smart query system considers query formulation, query relaxation, data mining techniques and the augmentation of semantic sensor web for answering query using reasoning.

## Figures and Tables

**Table 1.** Types of query processing based on answering techniques for sensor data.

**Class**	**Traditional query**		**Stream query**		**Smart query**
**Subclass**	**Sensor database**	**Sensor web**	**In-network query**	**Data stream management system (DSMS)**	**Sensor database, DSMS & Semantic Sensor Web**
**Examples**	Oracle, PostgreSQL, XML	Sensor Observation Service(SOS)	COUGAR [3,18] TinyDB [25]	Aurora [24] Nile [26]	Proposed
**Answer types**	Exact	Exact	Exact/Approximate	Exact/Approximate	Exact/Approximate
